# Diethyl 3,3′-[(3-fluoro­phen­yl)methyl­ene]bis­(1*H*-indole-2-carboxyl­ate)

**DOI:** 10.1107/S2414314620009128

**Published:** 2020-07-10

**Authors:** Hong Jiang, Yu-Long Li, Jin Zhou, Hong-Shun Sun, Qing-Yu Zhang, Xing-Hao Shi, Zhi-Yuan Zhang, Tian Ling

**Affiliations:** a Targeted MRI Contrast Agents Laboratory of Jiangsu Province, Nanjing Polytechnic Institute, Nanjing 210048, People’s Republic of China; b College of Chemistry and Molecular Engineering, Nanjing Tech University, Nanjing 211816, People’s Republic of China; University of Aberdeen, Scotland

**Keywords:** crystal structure, bis­indole, MRI, contrast agents

## Abstract

In the title compound, C_29_H_25_FN_2_O_4_, the mean planes of the indole ring systems are approximately perpendicular to one another [dihedral angle = 88.3 (4)°]. The benzene ring is twisted with respect to the indole ring systems by 49.8 (5) and 77.6 (3)°. In the crystal, pairs of N—H⋯O hydrogen bonds link the mol­ecules into the inversion dimers which are further linked into supra­molecular chains propagating along the [110] direction.

## Abstract 2

In the title compound, the indole ring systems are approximately perpendicular to one another with a dihedral angle of 88.3 (4)°.

## Graphical abstract

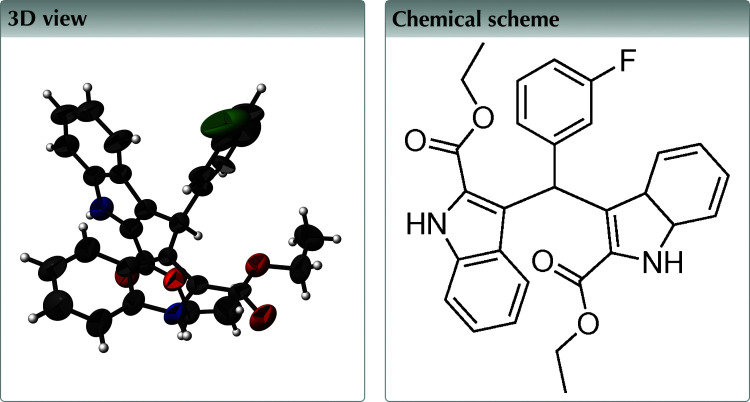

## Structure description

There are abundant bis­(indol­yl)methane derivatives in various terrestrial and marine natural resources (Sundberg, 1996 ▸). As part of our ongoing studies of bis­(indoyl)methane compounds, we now report the synthesis and crystal structure of the title compound.

**Figure 1:**
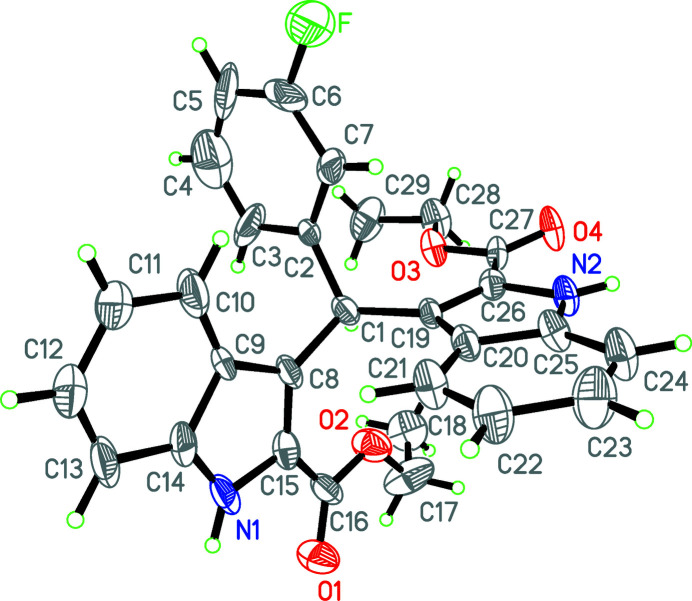
The mol­ecular structure of the title mol­ecule with displacement ellipsoids drawn at the 30% probability level.

The mol­ecular structure of the title compound is shown in Fig. 1 ▸. The indole ring systems are nearly perpendicular to one another [dihedral angle = 88.3 (4)°] while the benzene ring (C2–C7) is twisted with respect to the N1/C8–C15 and N2/C19–C26 indole ring systems with dihedral angles of 49.8 (5) and 77.6 (3)°, respectively. The carboxyl groups are approximately co-planar with their attached indole ring systems, the dihedral angles between the carboxyl groups and the mean planes of the N1/C8–C15 and N2/C19–C26 indole ring systems being 6.2 (5) and 6.4 (4)°, respectively.

**Figure 2:**
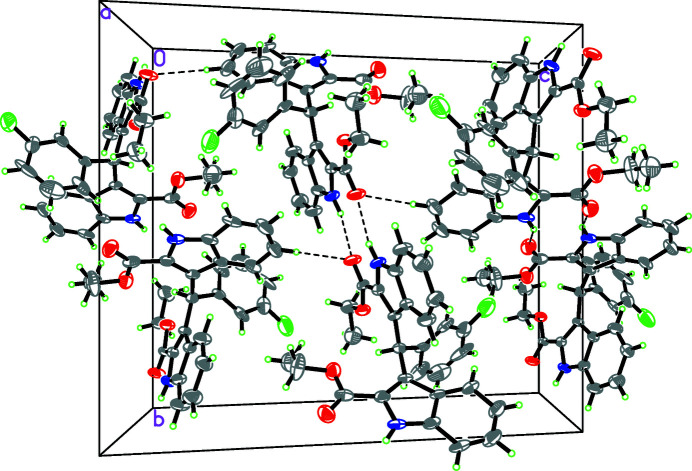
A packing diagram of the title compound. Hydrogen bonds are shown as dashed lines.

In the crystal, pairwise N1—H1*A*⋯O1^i^ and N2—H2*A*⋯O4^ii^ hydrogen bonds both generate 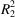 (8) loops; together these lead to [110] chains of mol­ecules. A weak C11—H11*A*⋯O4^iii^ inter­action also occurs, which links the chains into (001) sheets (Table 1 ▸ and Fig. 2 ▸).

Several similar structures have been reported previously, *viz*. diethyl 3,3′-(phenyl­methyl­ene)bis­(1*H*-indole-2-carboxyl­ate) (Sun *et al.*, 2012 ▸), dimethyl 3,3′-[(3-fluoro­phen­yl)methyl­ene]bis­(1*H*-indole-2-carboxyl­ate) (Lu *et al.*, 2014 ▸), dimethyl 3,3′-[(4-fluoro­phen­yl)methyl­ene]bis­(1*H*-indole-2-carboxyl­ate) (Sun *et al.*, 2015 ▸) and dimethyl 3,3′-[(2-fluoro­phen­yl)methyl­ene]bis­(1*H*-indole-2-carboxyl­ate) (Lu *et al.*, 2017 ▸). In these structures, the indole ring systems are also nearly perpendicular to one another, making dihedral angles of 82.0 (5), 87.8 (5), 84.0 (5) and 86.0 (5)°, respectively.

## Synthesis and crystallization

Ethyl indole-2-carboxyl­ate (1.88 g, 10 mmol) was dissolved in 20 ml of ethanol and 3-fluoro­benzaldehyde (0.62 g, 5 mmol) and concentrated HCl (0.5 ml) was added. The mixture was heated to reflux temperature for 2 h. After cooling, the white product was filtered off and washed thoroughly with ethanol (yield = 92%). Single crystals of the title compound suitable for X-ray analysis were obtained by slow evaporation of an ethanol solution.

## Refinement

Crystal data, data collection and structure refinement details are summarized in Table 2 ▸.

## Supplementary Material

Crystal structure: contains datablock(s) I, global. DOI: 10.1107/S2414314620009128/hb4349sup1.cif

Structure factors: contains datablock(s) I. DOI: 10.1107/S2414314620009128/hb4349Isup2.hkl

Supporting information file. DOI: 10.1107/S2414314620009128/hb4349Isup3.cml

CCDC reference: 2014000

Additional supporting information:  crystallographic information; 3D view; checkCIF report

## Figures and Tables

**Table 1:** Hydrogen-bond geometry (Å, °)

*D*—H⋯*A*	*D*—H	H⋯*A*	*D*⋯*A*	*D*—H⋯*A*
N1—H1*A*⋯O1^i^	0.86	2.16	2.918 (11)	147
N2—H2*A*⋯O4^ii^	0.86	2.08	2.904 (9)	159
C11—H11*A*⋯O4^iii^	0.93	2.58	3.498 (13)	171

Symmetry codes: (i) 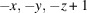 ; (ii) 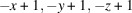 ; (iii) 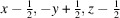 .

**Table 2:** Experimental details

Crystal data	
Chemical formula	C_29_H_25_FN_2_O_4_
*M*_r_	484.51
Crystal system, space group	Monoclinic, *P*2_1_/*n*
Temperature (K)	293
*a*, *b*, *c* (Å)	8.9960 (18), 15.921 (3), 18.297 (4)
β (°)	102.59 (3)
*V* (Å^3^)	2557.6 (9)
*Z*	4
Radiation type	Mo *K*α
μ (mm^−1^)	0.09
Crystal size (mm)	0.20 × 0.20 × 0.10

Data collection	
Diffractometer	Enraf–Nonius CAD-4
Absorption correction	ψ scan (North *et al.*, 1968 ▸)
*T*_min_, *T*_max_	0.982, 0.991
No. of measured, independent and observed [*I* > 2σ(*I*)] reflections	5000, 4685, 1424
*R*_int_	0.131
(sin θ/λ)_max_ (Å^−1^)	0.603

Refinement	
*R*[*F* ^2^ > 2σ(*F* ^2^)], *wR*(*F* ^2^), *S*	0.143, 0.306, 1.30
No. of reflections	4685
No. of parameters	319
H-atom treatment	H atoms treated by a mixture of independent and constrained refinement
Δρ_max_, Δρ_min_ (e Å^−3^)	0.56, −0.72

Computer programs: *CAD-4 EXPRESS* (Enraf–Nonius, 1994 ▸), *XCAD4* (Harms & Wocadlo, 1995 ▸) and *SHELXTL* (Sheldrick, 2008 ▸).

## full crystallographic data

### Crystal data

**Table:**

C_29_H_25_FN_2_O_4_	*F*(000) = 1016
*M**_r_* = 484.51	*D*_x_ = 1.258 Mg m^−^^3^
Monoclinic, *P*2_1_/*n*	Mo *K*α radiation, λ = 0.71073 Å
*a* = 8.9960 (18) Å	Cell parameters from 25 reflections
*b* = 15.921 (3) Å	θ = 9–12°
*c* = 18.297 (4) Å	µ = 0.09 mm^−^^1^
β = 102.59 (3)°	*T* = 293 K
*V* = 2557.6 (9) Å^3^	Block, colorless
*Z* = 4	0.20 × 0.20 × 0.10 mm

### Data collection

**Table:**

Enraf–Nonius CAD-4 diffractometer	1424 reflections with *I* > 2σ(*I*)
Radiation source: fine-focus sealed tube	*R*_int_ = 0.131
Graphite monochromator	θ_max_ = 25.4°, θ_min_ = 1.7°
ω/2θ scans	*h* = 0→10
Absorption correction: ψ scan (North *et al.*, 1968)	*k* = 0→19
*T*_min_ = 0.982, *T*_max_ = 0.991	*l* = −22→21
5000 measured reflections	3 standard reflections every 200 reflections
4685 independent reflections	intensity decay: 1%

### Refinement

**Table:**

Refinement on *F*^2^	Primary atom site location: structure-invariant direct methods
Least-squares matrix: full	Secondary atom site location: difference Fourier map
*R*[*F*^2^ > 2σ(*F*^2^)] = 0.143	Hydrogen site location: inferred from neighbouring sites
*wR*(*F*^2^) = 0.306	H atoms treated by a mixture of independent and constrained refinement
*S* = 1.30	*w* = 1/[σ^2^(*F*_o_^2^) + (0.060*P*)^2^] where *P* = (*F*_o_^2^ + 2*F*_c_^2^)/3
4685 reflections	(Δ/σ)_max_ = 0.009
319 parameters	Δρ_max_ = 0.56 e Å^−^^3^
0 restraints	Δρ_min_ = −0.71 e Å^−^^3^

### Special details

**Table:**

Geometry. All esds (except the esd in the dihedral angle between two l.s. planes) are estimated using the full covariance matrix. The cell esds are taken into account individually in the estimation of esds in distances, angles and torsion angles; correlations between esds in cell parameters are only used when they are defined by crystal symmetry. An approximate (isotropic) treatment of cell esds is used for estimating esds involving l.s. planes.
Refinement. Refinement of F^2^ against ALL reflections. The weighted R-factor wR and goodness of fit S are based on F^2^, conventional R-factors R are based on F, with F set to zero for negative F^2^. The threshold expression of F^2^ > 2sigma(F^2^) is used only for calculating R-factors(gt) etc. and is not relevant to the choice of reflections for refinement. R-factors based on F^2^ are statistically about twice as large as those based on F, and R- factors based on ALL data will be even larger. H atoms were positioned geometrically with N—H = 0.86 Å and C—H = 0.93–0.98 Å, and constrained to ride on their parent atoms. The constraint *U*_iso_(H) = 1.2*U*_eq_(C,N) or 1.5*U*_eq_(methyl C) was applied in all cases.

### Fractional atomic coordinates and isotropic or equivalent isotropic displacement parameters (Å^2^)

**Table:**

	*x*	*y*	*z*	*U*_iso_*/*U*_eq_	
F	0.5574 (10)	0.2828 (5)	0.1783 (4)	0.154 (4)	
O1	0.1733 (8)	0.0523 (4)	0.5601 (4)	0.080 (2)	
N1	0.0972 (9)	0.0284 (4)	0.4084 (5)	0.063 (2)	
H1A	0.0468	−0.0028	0.4327	0.076*	
C1	0.3860 (9)	0.1906 (5)	0.3962 (5)	0.045 (2)	
H1B	0.4561	0.1763	0.4434	0.054*	
N2	0.3321 (8)	0.4124 (4)	0.4463 (4)	0.054 (2)	
H2A	0.3634	0.4597	0.4668	0.064*	
O2	0.3615 (8)	0.1390 (4)	0.5478 (4)	0.078 (2)	
C2	0.4782 (11)	0.1845 (6)	0.3395 (6)	0.049 (3)	
O3	0.6571 (7)	0.2781 (3)	0.4797 (3)	0.0609 (19)	
C3	0.5727 (12)	0.1140 (8)	0.3428 (7)	0.094 (5)	
H3A	0.5770	0.0753	0.3812	0.112*	
O4	0.6412 (7)	0.4138 (4)	0.5044 (4)	0.071 (2)	
C4	0.659 (2)	0.1009 (11)	0.2907 (12)	0.143 (7)	
H4A	0.7197	0.0531	0.2945	0.172*	
C5	0.6584 (19)	0.1558 (11)	0.2334 (11)	0.129 (7)	
H5A	0.7151	0.1471	0.1972	0.155*	
C6	0.5694 (16)	0.2231 (10)	0.2335 (7)	0.092 (4)	
C7	0.4733 (12)	0.2417 (7)	0.2840 (6)	0.071 (3)	
H7A	0.4121	0.2893	0.2792	0.086*	
C8	0.2671 (10)	0.1236 (5)	0.3855 (6)	0.050 (3)	
C9	0.1838 (10)	0.0882 (5)	0.3150 (6)	0.051 (3)	
C10	0.1875 (12)	0.1021 (6)	0.2409 (7)	0.080 (4)	
H10A	0.2524	0.1420	0.2276	0.096*	
C11	0.0911 (12)	0.0547 (7)	0.1867 (6)	0.082 (4)	
H11A	0.0929	0.0626	0.1366	0.099*	
C12	−0.0061 (14)	−0.0033 (7)	0.2053 (7)	0.088 (4)	
H12A	−0.0678	−0.0341	0.1673	0.105*	
C13	−0.0162 (12)	−0.0175 (6)	0.2762 (7)	0.077 (3)	
H13A	−0.0844	−0.0565	0.2878	0.092*	
C14	0.0816 (11)	0.0295 (6)	0.3329 (7)	0.058 (3)	
C15	0.2065 (11)	0.0855 (6)	0.4391 (6)	0.057 (3)	
C16	0.2487 (12)	0.0887 (6)	0.5223 (7)	0.061 (3)	
C17	0.4070 (12)	0.1489 (9)	0.6278 (6)	0.108 (5)	
H17A	0.3662	0.2010	0.6427	0.129*	
H17B	0.3673	0.1029	0.6525	0.129*	
C18	0.5630 (13)	0.1500 (7)	0.6483 (7)	0.112	
H18A	0.5940	0.1565	0.7016	0.168*	
H18B	0.6015	0.1960	0.6241	0.168*	
H18C	0.6027	0.0982	0.6337	0.168*	
C19	0.3282 (9)	0.2778 (5)	0.4087 (5)	0.039 (2)	
C20	0.1817 (11)	0.3129 (5)	0.3856 (5)	0.049 (3)	
C21	0.0406 (12)	0.2819 (6)	0.3459 (6)	0.069 (3)	
H21A	0.0315	0.2268	0.3286	0.082*	
C22	−0.0847 (13)	0.3351 (7)	0.3330 (6)	0.077 (4)	
H22A	−0.1789	0.3152	0.3073	0.092*	
C23	−0.0710 (13)	0.4196 (7)	0.3585 (6)	0.089 (4)	
H23A	−0.1560	0.4544	0.3490	0.106*	
C24	0.0652 (12)	0.4502 (6)	0.3968 (6)	0.071 (3)	
H24A	0.0749	0.5056	0.4131	0.085*	
C25	0.1893 (11)	0.3958 (5)	0.4105 (5)	0.054 (3)	
C26	0.4231 (11)	0.3414 (5)	0.4456 (5)	0.044 (2)	
C27	0.5740 (11)	0.3489 (5)	0.4795 (5)	0.045 (2)	
C28	0.8156 (10)	0.2802 (6)	0.5124 (6)	0.075 (3)	
H28A	0.8324	0.2942	0.5651	0.089*	
H28B	0.8662	0.3218	0.4877	0.089*	
C29	0.8750 (12)	0.1962 (7)	0.5028 (6)	0.101 (4)	
H29A	0.9824	0.1948	0.5241	0.152*	
H29B	0.8573	0.1830	0.4504	0.152*	
H29C	0.8242	0.1557	0.5276	0.152*	

### Atomic displacement parameters (Å^2^)

**Table:**

	*U*^11^	*U*^22^	*U*^33^	*U*^12^	*U*^13^	*U*^23^
F	0.256 (10)	0.129 (7)	0.088 (6)	−0.081 (7)	0.063 (6)	−0.033 (5)
O1	0.085 (6)	0.085 (5)	0.073 (6)	−0.020 (4)	0.022 (4)	0.013 (4)
N1	0.076 (6)	0.026 (4)	0.088 (7)	−0.012 (4)	0.016 (6)	−0.001 (5)
C1	0.050 (6)	0.033 (5)	0.053 (7)	−0.012 (5)	0.011 (5)	0.000 (5)
N2	0.058 (5)	0.031 (4)	0.065 (6)	−0.009 (4)	0.001 (5)	−0.015 (4)
O2	0.081 (5)	0.091 (6)	0.060 (5)	−0.032 (5)	0.014 (4)	−0.005 (4)
C2	0.057 (7)	0.035 (6)	0.065 (8)	−0.025 (5)	0.033 (6)	−0.021 (5)
O3	0.068 (5)	0.036 (4)	0.072 (5)	0.002 (4)	0.001 (4)	−0.004 (3)
C3	0.068 (8)	0.109 (11)	0.117 (12)	−0.005 (8)	0.049 (8)	−0.068 (9)
O4	0.079 (5)	0.030 (4)	0.093 (5)	−0.004 (4)	−0.008 (4)	−0.016 (4)
C4	0.132 (15)	0.105 (14)	0.18 (2)	−0.017 (12)	0.016 (15)	−0.038 (13)
C5	0.132 (14)	0.101 (13)	0.169 (19)	−0.033 (12)	0.065 (14)	−0.106 (13)
C6	0.113 (11)	0.097 (11)	0.066 (10)	−0.081 (9)	0.022 (9)	−0.013 (9)
C7	0.079 (8)	0.067 (8)	0.077 (9)	−0.039 (7)	0.036 (7)	−0.046 (7)
C8	0.062 (7)	0.025 (5)	0.061 (7)	−0.011 (5)	0.013 (6)	−0.002 (5)
C9	0.063 (7)	0.025 (5)	0.069 (8)	−0.017 (5)	0.023 (6)	−0.012 (6)
C10	0.104 (9)	0.053 (7)	0.077 (9)	−0.029 (6)	0.009 (8)	−0.024 (7)
C11	0.097 (9)	0.081 (8)	0.070 (9)	−0.023 (7)	0.021 (7)	−0.029 (7)
C12	0.105 (10)	0.074 (9)	0.084 (10)	−0.019 (8)	0.023 (9)	−0.040 (8)
C13	0.084 (8)	0.034 (6)	0.112 (10)	−0.010 (6)	0.019 (8)	−0.028 (7)
C14	0.063 (7)	0.032 (6)	0.076 (9)	0.006 (5)	0.006 (7)	−0.015 (6)
C15	0.069 (7)	0.031 (6)	0.067 (8)	0.007 (5)	0.010 (7)	−0.003 (6)
C16	0.053 (7)	0.039 (6)	0.094 (10)	−0.010 (6)	0.022 (7)	0.006 (6)
C17	0.062 (8)	0.200 (14)	0.058 (9)	−0.006 (8)	0.008 (7)	−0.030 (9)
C18	0.112	0.112	0.112	0.000	0.024	0.000
C19	0.041 (6)	0.026 (5)	0.049 (6)	−0.005 (4)	0.010 (5)	−0.006 (4)
C20	0.065 (7)	0.032 (5)	0.047 (6)	−0.009 (5)	0.008 (6)	−0.004 (5)
C21	0.072 (8)	0.042 (6)	0.085 (9)	−0.001 (6)	0.003 (7)	−0.010 (6)
C22	0.089 (9)	0.061 (8)	0.070 (8)	−0.002 (7)	−0.008 (7)	−0.015 (6)
C23	0.086 (9)	0.072 (8)	0.098 (10)	0.028 (7)	−0.002 (8)	−0.001 (8)
C24	0.068 (8)	0.039 (6)	0.097 (9)	−0.002 (6)	−0.003 (7)	−0.013 (6)
C25	0.054 (7)	0.036 (6)	0.063 (7)	−0.001 (5)	−0.007 (6)	0.004 (5)
C26	0.053 (6)	0.036 (5)	0.044 (6)	0.003 (5)	0.009 (5)	−0.001 (5)
C27	0.060 (7)	0.030 (5)	0.042 (6)	0.006 (5)	0.001 (5)	−0.008 (5)
C28	0.053 (7)	0.056 (7)	0.100 (9)	0.015 (6)	−0.016 (7)	0.001 (6)
C29	0.097 (9)	0.099 (9)	0.104 (10)	0.053 (8)	0.012 (8)	−0.007 (8)

### Geometric parameters (Å, º)

**Table:**

F—C6	1.374 (13)	C11—C12	1.365 (13)
O1—C16	1.216 (10)	C11—H11A	0.9300
N1—C14	1.356 (11)	C12—C13	1.339 (13)
N1—C15	1.367 (10)	C12—H12A	0.9300
N1—H1A	0.8600	C13—C14	1.418 (12)
C1—C2	1.464 (11)	C13—H13A	0.9300
C1—C8	1.492 (10)	C15—C16	1.488 (13)
C1—C19	1.517 (10)	C17—C18	1.371 (12)
C1—H1B	0.9800	C17—H17A	0.9700
N2—C25	1.335 (10)	C17—H17B	0.9700
N2—C26	1.398 (9)	C18—H18A	0.9600
N2—H2A	0.8600	C18—H18B	0.9600
O2—C16	1.297 (10)	C18—H18C	0.9600
O2—C17	1.440 (11)	C19—C26	1.399 (10)
C2—C7	1.359 (13)	C19—C20	1.409 (11)
C2—C3	1.401 (13)	C20—C25	1.394 (11)
O3—C27	1.351 (9)	C20—C21	1.407 (11)
O3—C28	1.421 (9)	C21—C22	1.389 (12)
C3—C4	1.370 (18)	C21—H21A	0.9300
C3—H3A	0.9300	C22—C23	1.420 (12)
O4—C27	1.234 (9)	C22—H22A	0.9300
C4—C5	1.363 (19)	C23—C24	1.361 (12)
C4—H4A	0.9300	C23—H23A	0.9300
C5—C6	1.339 (18)	C24—C25	1.392 (11)
C5—H5A	0.9300	C24—H24A	0.9300
C6—C7	1.426 (15)	C26—C27	1.370 (11)
C7—H7A	0.9300	C28—C29	1.465 (11)
C8—C15	1.364 (11)	C28—H28A	0.9700
C8—C9	1.457 (12)	C28—H28B	0.9700
C9—C10	1.382 (12)	C29—H29A	0.9600
C9—C14	1.400 (11)	C29—H29B	0.9600
C10—C11	1.389 (12)	C29—H29C	0.9600
C10—H10A	0.9300		
			
C14—N1—C15	108.4 (9)	C8—C15—C16	131.9 (10)
C14—N1—H1A	125.8	O1—C16—O2	125.4 (11)
C15—N1—H1A	125.8	O1—C16—C15	121.1 (10)
C2—C1—C8	111.1 (7)	O2—C16—C15	113.2 (10)
C2—C1—C19	115.7 (7)	C18—C17—O2	109.1 (10)
C8—C1—C19	114.4 (7)	C18—C17—H17A	109.9
C2—C1—H1B	104.7	O2—C17—H17A	109.9
C8—C1—H1B	104.7	C18—C17—H17B	109.9
C19—C1—H1B	104.7	O2—C17—H17B	109.9
C25—N2—C26	109.7 (7)	H17A—C17—H17B	108.3
C25—N2—H2A	125.1	C17—C18—H18A	109.5
C26—N2—H2A	125.1	C17—C18—H18B	109.5
C16—O2—C17	117.4 (9)	H18A—C18—H18B	109.5
C7—C2—C3	119.2 (11)	C17—C18—H18C	109.5
C7—C2—C1	123.7 (10)	H18A—C18—H18C	109.5
C3—C2—C1	117.1 (10)	H18B—C18—H18C	109.5
C27—O3—C28	119.0 (7)	C26—C19—C20	106.9 (7)
C4—C3—C2	121.6 (15)	C26—C19—C1	122.8 (8)
C4—C3—H3A	119.2	C20—C19—C1	130.2 (7)
C2—C3—H3A	119.2	C25—C20—C21	118.4 (9)
C3—C4—C5	122.0 (19)	C25—C20—C19	107.5 (8)
C3—C4—H4A	119.0	C21—C20—C19	134.1 (8)
C5—C4—H4A	119.0	C22—C21—C20	118.8 (9)
C6—C5—C4	114.4 (18)	C22—C21—H21A	120.6
C6—C5—H5A	122.8	C20—C21—H21A	120.6
C4—C5—H5A	122.8	C21—C22—C23	120.8 (10)
C5—C6—F	120.2 (17)	C21—C22—H22A	119.6
C5—C6—C7	127.9 (14)	C23—C22—H22A	119.6
F—C6—C7	111.8 (15)	C24—C23—C22	120.8 (10)
C2—C7—C6	114.8 (11)	C24—C23—H23A	119.6
C2—C7—H7A	122.6	C22—C23—H23A	119.6
C6—C7—H7A	122.6	C23—C24—C25	117.8 (9)
C15—C8—C9	104.8 (8)	C23—C24—H24A	121.1
C15—C8—C1	127.7 (9)	C25—C24—H24A	121.1
C9—C8—C1	127.4 (9)	N2—C25—C24	127.8 (9)
C10—C9—C14	119.6 (10)	N2—C25—C20	108.8 (8)
C10—C9—C8	133.6 (9)	C24—C25—C20	123.4 (9)
C14—C9—C8	106.8 (9)	C27—C26—N2	116.7 (8)
C11—C10—C9	117.9 (10)	C27—C26—C19	136.2 (8)
C11—C10—H10A	121.0	N2—C26—C19	107.1 (7)
C9—C10—H10A	121.0	O4—C27—O3	118.1 (8)
C12—C11—C10	121.6 (11)	O4—C27—C26	126.7 (8)
C12—C11—H11A	119.2	O3—C27—C26	114.9 (8)
C10—C11—H11A	119.2	O3—C28—C29	106.7 (8)
C13—C12—C11	122.5 (12)	O3—C28—H28A	110.4
C13—C12—H12A	118.7	C29—C28—H28A	110.4
C11—C12—H12A	118.7	O3—C28—H28B	110.4
C12—C13—C14	117.2 (11)	C29—C28—H28B	110.4
C12—C13—H13A	121.4	H28A—C28—H28B	108.6
C14—C13—H13A	121.4	C28—C29—H29A	109.5
N1—C14—C9	108.6 (9)	C28—C29—H29B	109.5
N1—C14—C13	130.4 (11)	H29A—C29—H29B	109.5
C9—C14—C13	121.0 (11)	C28—C29—H29C	109.5
N1—C15—C8	111.4 (9)	H29A—C29—H29C	109.5
N1—C15—C16	116.3 (10)	H29B—C29—H29C	109.5
			
C8—C1—C2—C7	110.8 (9)	C17—O2—C16—O1	3.8 (15)
C19—C1—C2—C7	−21.8 (13)	C17—O2—C16—C15	177.6 (9)
C8—C1—C2—C3	−67.4 (11)	N1—C15—C16—O1	−11.3 (14)
C19—C1—C2—C3	159.9 (8)	C8—C15—C16—O1	176.3 (10)
C7—C2—C3—C4	−0.8 (16)	N1—C15—C16—O2	174.6 (8)
C1—C2—C3—C4	177.5 (11)	C8—C15—C16—O2	2.1 (15)
C2—C3—C4—C5	1 (2)	C16—O2—C17—C18	138.6 (11)
C3—C4—C5—C6	1 (2)	C2—C1—C19—C26	−72.7 (11)
C4—C5—C6—F	−179.3 (12)	C8—C1—C19—C26	156.2 (8)
C4—C5—C6—C7	−3 (2)	C2—C1—C19—C20	104.3 (11)
C3—C2—C7—C6	−0.4 (13)	C8—C1—C19—C20	−26.8 (14)
C1—C2—C7—C6	−178.6 (8)	C26—C19—C20—C25	−1.7 (10)
C5—C6—C7—C2	2.2 (17)	C1—C19—C20—C25	−179.0 (9)
F—C6—C7—C2	179.2 (8)	C26—C19—C20—C21	179.0 (10)
C2—C1—C8—C15	149.4 (10)	C1—C19—C20—C21	1.6 (18)
C19—C1—C8—C15	−77.3 (12)	C25—C20—C21—C22	0.2 (15)
C2—C1—C8—C9	−34.5 (13)	C19—C20—C21—C22	179.5 (10)
C19—C1—C8—C9	98.8 (11)	C20—C21—C22—C23	0.8 (16)
C15—C8—C9—C10	177.8 (11)	C21—C22—C23—C24	−0.5 (18)
C1—C8—C9—C10	1.0 (17)	C22—C23—C24—C25	−0.6 (17)
C15—C8—C9—C14	−1.5 (10)	C26—N2—C25—C24	−177.8 (10)
C1—C8—C9—C14	−178.2 (8)	C26—N2—C25—C20	0.5 (11)
C14—C9—C10—C11	−1.9 (15)	C23—C24—C25—N2	179.7 (10)
C8—C9—C10—C11	178.9 (10)	C23—C24—C25—C20	1.7 (16)
C9—C10—C11—C12	0.9 (16)	C21—C20—C25—N2	−179.8 (8)
C10—C11—C12—C13	0.7 (19)	C19—C20—C25—N2	0.7 (11)
C11—C12—C13—C14	−1.2 (18)	C21—C20—C25—C24	−1.4 (15)
C15—N1—C14—C9	0.3 (10)	C19—C20—C25—C24	179.1 (9)
C15—N1—C14—C13	−179.8 (9)	C25—N2—C26—C27	179.2 (8)
C10—C9—C14—N1	−178.7 (9)	C25—N2—C26—C19	−1.5 (10)
C8—C9—C14—N1	0.7 (10)	C20—C19—C26—C27	−179.0 (11)
C10—C9—C14—C13	1.4 (14)	C1—C19—C26—C27	−1.4 (16)
C8—C9—C14—C13	−179.2 (8)	C20—C19—C26—N2	1.9 (10)
C12—C13—C14—N1	−179.8 (10)	C1—C19—C26—N2	179.5 (8)
C12—C13—C14—C9	0.1 (15)	C28—O3—C27—O4	3.8 (13)
C14—N1—C15—C8	−1.3 (11)	C28—O3—C27—C26	178.8 (8)
C14—N1—C15—C16	−175.3 (8)	N2—C26—C27—O4	−7.4 (14)
C9—C8—C15—N1	1.7 (10)	C19—C26—C27—O4	173.6 (10)
C1—C8—C15—N1	178.5 (8)	N2—C26—C27—O3	178.1 (7)
C9—C8—C15—C16	174.4 (9)	C19—C26—C27—O3	−1.0 (16)
C1—C8—C15—C16	−8.8 (16)	C27—O3—C28—C29	−178.8 (8)

### Hydrogen-bond geometry (Å, º)

**Table:**

*D*—H···*A*	*D*—H	H···*A*	*D*···*A*	*D*—H···*A*
N1—H1*A*···O1^i^	0.86	2.16	2.918 (11)	147
N2—H2*A*···O4^ii^	0.86	2.08	2.904 (9)	159
C11—H11*A*···O4^iii^	0.93	2.58	3.498 (13)	171

Symmetry codes: (i) −*x*, −*y*, −*z*+1; (ii) −*x*+1, −*y*+1, −*z*+1; (iii) *x*−1/2, −*y*+1/2, *z*−1/2.
